# The relationship between physical exercise and subjective well-being in Chinese older people: The mediating role of the sense of meaning in life and self-esteem

**DOI:** 10.3389/fpsyg.2022.1029587

**Published:** 2022-11-10

**Authors:** Rui Chen, Yong-Feng Liu, Gao-Duan Huang, Peng-Cheng Wu

**Affiliations:** ^1^Institute of Sports Training, Chengdu Sport University, Chengdu, China; ^2^School of Chinese and Literature, Yunnan University, Kunming, China; ^3^School of Electronic and Electrical Engineering, University of Leeds, Leeds, United Kingdom

**Keywords:** physical exercise, subjective well-being of older people, chain mediation, sense of meaning in life, self-esteem

## Abstract

**Purpose:**

This study explores the relationship between physical exercise and older people’s subjective well-being and the mediating role of a sense of meaning in life and self-esteem by using a structural equation modeling (SEM) approach, in order to provide some suggestions for improving older people’s subjective well-being.

**Methods:**

In this study, a cross-sectional survey was conducted offline using a simple random method of collection, and the Physical Activity Rating Scale (PARS-3), the Subjective Well-being Scale (SWB), the Meaningfulness of Life Scale (MLQ), and the Self-Esteem Scale (SES) were applied to 419 older adults who participated in physical exercise from Chengdu (Qingyang District, Wuhou District, and Chenghua District), Sichuan Province, China, with the voluntary participation of the subjects. 197 males and 222 females, with a mean age of 72.49 (SD = 1.57). The study used SPSS 25.0 and Process 3.5 plug-in for statistical processing of the data, Cronbach’s alpha coefficient for intra-variate consistency testing, Harman’s one-way test for common method bias testing and multiple covariance diagnosis, and finally regression analysis and Bootstrap sampling test for significance of mediating effects.

**Results:**

Physical exercise was able to have a positive effect on the level of subjective well-being of older adults (*β* = 0.0305; 95% confidence interval (CI): 0.0226, 0.0384; *p* < 0.05), and a mediation analysis of sense of meaning in life and self-esteem revealed that they were able to have independent and chained mediation effects, with four pathways: first, physical exercise directly affected subjective well-being of older adults (*β* = 0.0149; 95% CI: 0.0072, 0.0226; *p* < 0.05; *β* = 0.0149; 95% CI: 0.0072, 0.0226; *p* < 0.05); secondly, sense of meaning in life mediated the relationship between physical exercise and subjective well-being of older adults (*β* = 0.0075; 95% CI: 0.0041, 0.0115; *p* < 0.05); thirdly, self-esteem mediated the relationship between physical exercise and subjective well-being of older adults (*β* = 0.0075; 95% CI: 0.0041, 0.0115; *p* < 0.05). (β = 0.0061; 95% CI: 0.0034, 0.0094; *p* < 0.05); fourth, a chain mediating effect of sense of meaning in life and self-esteem in the relationship between physical exercise and subjective well-being in older adults (*β* = 0.0021; 95% CI: 0.0010, 0.0035; *p* < 0.05).

**Conclusion and prospects:**

As indicated by the results, physical exercise can enhance the subjective well-being of older adults through sense of meaning in life and self-esteem, therefore, in order to be able to enhance the subjective well-being of older adults, enhancing the level of sense of meaning in life and self-esteem of older adults is an effective means.

## Introduction

As the number of older people continues to increase, the economic and social development and the system will face serious challenges (Gao, 2022). At the same time, the overall assessment of the quality of life in old age will tend to be subjectively lowered due to the negative realities of retirement, the decline in one’s own functioning, reduced care from relatives, and the death of family members (Wu, 2008; Spesivtseva, 2015). As an important indicator of the quality of life, low subjective well-being can lead to depression, anxiety, and other psychological disorders in older people (Diener et al., 1997), which can seriously affect life satisfaction and marital satisfaction. According to Wang (2017)and Jiang et al. (2009), physical exercise is a beneficial physical exercise with a certain intensity, frequency, and duration, which can effectively reduce the risk of cardiovascular disease in the elderly (Li and Gao, 2019). It is able to enhance their mental cognition (Liu et al., 2016), improve memory and executive function (Cai et al., 2021), and boost their quality of life and body confidence (Tokuda and Mori, 2021). Based on the research, it was found that physical exercise that enhances well-being is an essential option for achieving a healthy lifestyle in older adults (Yang et al., 2019). Regular physical exercise can not only significantly improve cognitive function, quality of life, and well-being in older adults (Silverstein and Parker, 2002), but also improve their body’s internal environment. However, due to the low awareness and low proportion of physical exercise among the current Chinese elderly population (Gao and Zhang, 2020), it has become an important reason for the deterioration of subjective well-being and quality of life in the process of the paradigm shift of the Chinese population death. The relationship between physical exercise and subjective well-being in university students has been studied, and the mechanisms underlying and mediating the relationship between physical exercise and subjective well-being in older people have been less studied (Zhou and Zhou, 2022). Based on this, this study explores how physical exercise affects subjective well-being in older people and the possible mechanisms underlying the relationship between the two, in order to provide evidence for improving the mental health of older people in the context of increasing aging.

### Theoretical basis, research aims, and hypothesis

This study aims to build on existing research to explore the relationship between physical exercise and subjective well-being of older people and its underlying mechanisms. The study shows that Subjective well-being is commonly used to assess personal life satisfaction, the presence or absence of anxiety and depression (Diener, 1994), and other factors such as long-term happiness, pleasant emotions, and life satisfaction. Because many intrinsic elements (such as high quality of life, positive emotions, and so on) are closely related to life experiences, they are more frequently used for individual life evaluations. For older people entering their later years of life, searching for a better life experience is a value and the end of life gives more meaning. Therefore, the question of how to improve subjective well-being in older people has arisen and received much attention. According to Zhai and Wang (2018), social support, the external environment, and mental health all predict subjective well-being in older people. Meanwhile Zhan and Zhao (2018), proposed that older people’s subjective well-being is closely related to the community environment, the number of community services, and community capital. At present, there is a wealth of research on the relationship between physical exercise and subjective well-being, but most of the studies focus on the relationship between physical exercise and subjective well-being of university students or middle-aged people, while few studies have explored the interrelationship between physical exercise and subjective well-being of older people and their underlying mechanisms. Therefore, it is the starting point of this study to enrich the theoretical system of the relationship between physical exercise and subjective well-being of the elderly, and then to provide evidence to enhance the subjective well-being of the elderly by discovering other factors that may exist between the two.

### The effect of physical exercise on subjective well-being of older people

Physical exercise is defined as a conscious and purposeful social activity undertaken by individuals or groups to achieve physical and mental pleasure, enhance social interaction, and experience cultural life (Lu, 2011), Quality of life, life satisfaction, and physical fitness have all been shown in studies to be important indicators of subjective well-being (Xu and Xia, 2014). Simultaneously, Zheng et al. (2016) suggest that physical exercise can promote physical and mental health, strengthen self-confidence, and ultimately achieve a sense of meaning in life. In response to this conclusion, Luo et al. (2008) further concluded that physical exercise for the elderly should be done over a long period of time in order to sustain the positive effects on quality of life and subjective well-being. In addition, Stathi et al. (2010) suggest that physical exercise, with the exception of material well-being, can influence all aspects of subjective well-being in older people and promote psychological well-being by maintaining a busy life course, subjectively creating an active lifestyle, maintaining a good mental state, and a better attitude toward life. At the same time, McAuley and Rudolph (1995) stated that physical exercise is closely related to subjective well-being, it is unaffected by age or gender, but only by the physical condition of the subjects and the subjective well-being measurement methods. Wang et al. (2018) believe that physical exercise can improve social and economic status and behavior, thereby improving subjective well-being. There are also studies on physical exercise intervention for elderly medical patients. Following the experiment, it was discovered that patients’ quality of life and physical health had significantly improved, implying that patients’ subjective well-being had improved during treatment (Oldervoll et al., 2004). Therefore, based on the above analysis, this study proposes the hypothesis H1: Physical exercise positively predicts subjective well-being in older adults.

### The mediating role of a sense of meaning in life

Research on the relationship between physical exercise and subjective well-being in older adults have shown sophisticated mediating mechanisms. Physical exercise may affect elder’s subjective well-being by enhancing their sense of meaning in life, Normally (Ding et al., 2016). A subjective psychological assessment of a person’s future life objectives, directions, missions, and meanings is done through an independent judgment of the worth and significance of their current existence (Steger et al., 2008). It has a direct impact on life satisfaction, physical and mental health, and filial piety (Xie et al., 2012; Xu et al., 2020; Li et al., 2021). It has become one of the hottest scientific subjects in recent years. The final stage of life, old age, has a special significance. Exploring the connection between the meaning of life and how physical exercise affects the subjective well-being of the elderly is particularly crucial, because when people move from an active existence to a pleasant old age, they must modify their expectations for the future. According to study, a person’s subjective well-being as well as their physical and mental health may be enhanced by having a better sense of meaning in life (Ye et al., 2019). A higher sense of meaning in life can not only improve physical and mental health but also life satisfaction, experience, and the prevalence of anxiety and depression, according to other linked studies (Nell, 2014; Bamonti et al., 2016). Simultaneously, some researchers argue that the motivation (meaning pursuit) and cognition (meaning experience) contained in sense of meaning life cannot all predict subjective well-being. Only one aspect of quality of life clearly demonstrates that it is more important in the process of improving well-being and reducing depression and anxiety symptoms. This is because it is unclear how much each aspect of the sense of meaning in life affects the subjective well-being of the elderly, and it is necessary to figure out how a sense of meaning in life can act as an intermediary variable between the former and the latter (Steger et al., 2009; Hallford et al., 2018). The current study’s findings are inconclusive as to whether the meaningful life pursuit component affects well-being. Given the uncertainty regarding the extent to which the dimensions of sense of meaning in life affect subjective well-being in older adults, as well as the need to understand how sense of meaning in life as a mediating variable plays a role between the two before and after, this study proposes hypothesis H2: based on the preceding research, sense of meaning in life may play a mediating role in the relationship between physical exercise and subjective well-being in older adults.

### The mediating role of self-esteem

Self-esteem is an important outcome variable for physical exercise and may be another important internal factor (Zhu and Li, 2022), in the relationship between physical exercise and subjective well-being in older adults (Duman et al., 2016). It is a critical outcome variable in physical exercise. Individuals or organizations, according to the Social Information Processing Theory (SIPT) hypothesis, obtain relevant information from the immediate environment in which they participate in physical exercise, and interpret expectations about the occurrence and outcomes of physical exercise events, which leads to changes in self-worth judgments of individuals or members within the organization, and further influences levels of self-esteem. Physical exercise, according to research, can help female athletes improve positive psychosocial factors such as self-confidence, self-esteem, and decisiveness (Ferguson et al., 2019). Physical exercise, on the other hand, as an organized activity, is likely to have an impact on the self-esteem levels of older people. In addition, self-esteem is a strong predictor of subjective well-being and an important factor in the psychological well-being of older people, with high self-esteem implying positive attitudes toward values (Cheng and Furnham, 2003). This suggests that in older adults, self-esteem is a likely mediator of physical exercise as a predictor of subjective well-being. Current research on the effects of physical exercise on the subjective well-being of older people, however, has primarily focused on external variables such as place attachment, social interaction, and physical health status. Few studies have examined the relationship between physical exercise and subjective well-being in older adults, with psychological mechanisms such as self-esteem serving as moderators. As a result, the following hypothesis H3 is proposed in this study: Self-esteem may play a mediating role in the relationship between physical exercise and subjective well-being in older adults.

### The chain mediating role of sense of meaning in life and self-esteem

Furthermore, the sense of meaning in life and self-esteem are important factors influencing quality of life and physical and mental health, they are both conditioned by physical exercise (Zhang et al., 2021), which implies that a sense of meaning in life and self-esteem may play a chain mediating role in the effect of physical exercise level on subjective well-being of older adults. To begin, Wong (2010) propose that the meaning of life influences an individual’s internal psychological perceptions, such as reducing the unpleasantness of life processes and mitigating hardships by increasing levels of self-esteem. Secondly, research indicates that purpose, correctness, self-efficacy, and self-worth satisfaction are the four important sources for the formation of a sense of meaning in life (Baumeister, 1991), with self-efficacy and self-worth being subordinate to self-esteem. Given the similarity of the two connotation factors, it is reasonable to conclude that a sense of meaning life is an important factor influencing individual self-esteem. The sense of meaning in life positively predicts self-esteem, that is, the higher the sense of meaning in life, the higher the level of self-esteem reflected by individuals (Cao et al., 2019). Existing research confirms that physical exercise can affect older people’s subjective well-being both directly and indirectly *via* the sense of meaning in life or self-esteem, but no literature exists on whether the sense of meaning in life and self-esteem play a chain mediating role in the relationship between physical exercise and older people’s subjective well-being. Given the importance of improving older adults’ psychological health and promoting the growth of well-being, as well as the importance of investigating physical exercise to promote subjective well-being among older adults, this study proposes hypothesis H4: A sense of meaning in life and self-esteem may play a chain mediating effect in the relationship between physical exercise and subjective well-being of older adults.

**Figure 1:**
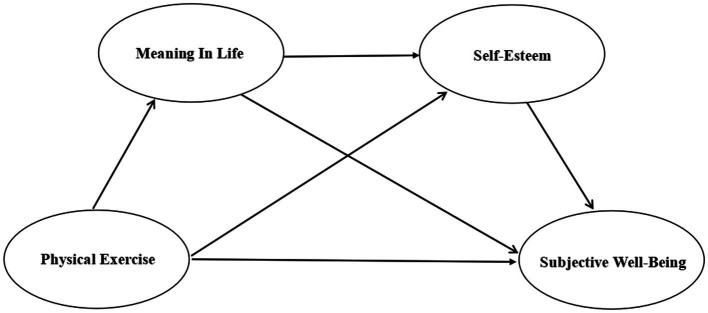
Research hypothesis model.

In summary, following the literature review and theoretical confirmation, this study suggests that a sense of meaning in life and self-esteem may be mediating variables between physical exercise and subjective well-being in older people (Figure 1).

## Research methodology

### Study subjects

An offline survey was conducted between 11 September 2021 and 19 December 2021 through offline face-to-face communication using a simple random sampling method among 450 older people aged 60 years and above from Chengdu (Qingyang District, Wuhou District, and Chenghua District), the capital of Sichuan Province, China, who had experienced physical exercise in the past month. The questionnaires were made clear to the subjects before the survey and the corresponding terms and conditions, and were completed independently or guided anonymously with the consent of the subjects and their families, and dictated by the investigator to ensure that the subjects could fully understand the meaning of each question item. After completing the overall survey, the data were checked and 31 questionnaires that were not completed in a standard way or missing answers were excluded, resulting in a total of 419 valid questionnaires with an effective rate of 93.11%. One hundred ninety-seven males and 222 females were included in the sample, with a mean age of 72.49 (SD = 1.57). The Ethics Review Committee (approval no. CTYLL2022001) of Chengdu Sports Institute, China, approved and supervised the entire content of this study. See Table 1 for basic information about the older people who participated in the survey.

**Table 1:** Subjects’ basic information characteristics (*N* = 419).

Category	Frequency	Percent
Gender		
Male	197	47.1
Female	222	52.9
District		
Qingyang district	91	21.71
Wuhou district	186	44.39
Chenghua district	142	33.89
Education level		
junior college and above	97	23.15
middle school	274	65.39
Other	48	11.46

### Measuring tools

#### Physical exercise rating scale

This study used the Physical Activity Rating Scale (PARS-3) revised by Liang (1994), which measures physical exercise rating from three perspectives: frequency, time, and intensity. Physical Activity Rating = Frequency x Time x Intensity, and all three dimensions are scored on a 5-point Likert scale. The higher the score, the more intense, more frequent, and longer the exercise, and conversely, the less intense, less frequent, and shorter the exercise. The physical exercise scale scores range from 0 to 100, with scores ≤19 being the low-level exercise group, scores between 20 and 42 being the medium-level exercise group, and scores ≥ 43 being the high-level exercise group. According to statistics, the physical exercise level of all older people was 33.69 (SD = 31.39). The overall Cronbach’s alpha coefficient for the PARS-3 was 0.836 (overall values range from 0 to 1; a coefficient of < 0.6 indicates that the scale has low internal consistency; a coefficient of 0.7–0.8 indicates that the scale has good reliability; a coefficient of 0.8–0.8 indicates that the scale has good reliability; and a coefficient between 0.8 and 0.9 indicates very good reliability).

#### Subjective well-being questionnaire

The Subjective Well-Being Scale (SWB) was developed by Campbell (1976), The SWB consists of eight general affective index items (weighted at 1.0) and one life satisfaction item (weighted at 1.1) on a 7-point Likert scale, with total subjective well-being scores ranging from 2.1 (least happy) to 14.7 (happiest), with higher total scores indicating higher levels of subjective well-being and vice versa. Validation of the measurement model resulted in a fit of *X*^2^/*df* = 0.923, Goodness-of-Fit Index (GFI) = 0.988, Comparative Fit Index (CFI) = 1, Tucker-Lewis Index (TLI) = 1, Root Mean Square Error of Approximation (RMSEA) = 0.000, and Standardized Root Mean Square Residual (SRMR) = 0.014 (Wei, 2008). The overall Cronbach’s alpha coefficient for the SWB was 0.929 (overall values range from 0 to 1; a coefficient of <0.6 indicates that the scale has low internal consistency; a coefficient of 0.7–0.8 indicates that the scale has good reliability; a coefficient of 0.8–0.8 indicates that the scale has good reliability; and a coefficient between 0.8 and 0.9 indicates very good reliability).

#### The sense of meaning in life scale

The Meaningful Life Scale (MLQ) was developed by Steger and revised by Wang and Dai (2008). The MLQ contains two dimensions, the presence of meaning (MLQ-P) and the search of meaning (MLQ-S), with a total of 10 questions. The items ranged from “1 = not at all agree” to “7 = completely agree,” with a higher total score indicating a stronger sense of meaning in life, and vice versa. *X*^2^/*df* = 0.922, GFI = 0.985, CFI = 1.001, TLI = 1.001, RMSEA = 0.000, and SRMR = 0.015.0.015, indicating good scale construct validity. The overall Cronbach’s alpha coefficient for the MLQ was 0.934 (overall values range from 0 to 1; a coefficient of < 0.6 indicates that the scale has low internal consistency; a coefficient of 0.7–0.8 indicates that the scale has good reliability; a coefficient of 0.8–0.8 indicates that the scale has good reliability; and a coefficient between 0.8 and 0.9 indicates very good reliability).

#### The self-esteem scale

In this study, the Rosenberg Self-Esteem Scale (SES) was used to measure the self-esteem of older people (Tian, 2006). The 10-item scale consists of two sections: self-affirmation, such as “I think I have a lot of good qualities,” and self-denial, such as “I often think I am useless” The scale is scored on a 4-point Likert scale, with questions 3, 5, 8, 9, and 10 being scored inversely, with each question ranging from “1 = very unlikely” to “4 = very likely,” with higher scores indicating higher levels of self-esteem. *X*^2^/*df* = 1.303, GFI = 0.979, CFI = 0.995, TLI = 0.994, RMSEA = 0.027, SRMR = 0.021. This indicates good construct validity of the SES. The Cronbach’s alpha coefficients for the self-affirmation and self-denial subscales were 0.852 and 0.850, respectively (overall values range from 0–1; a coefficient of < 0.6 indicates that the scale has low internal consistency; a coefficient of 0.7–0.8 indicates that the scale has good reliability; a coefficient of 0.8–0.8 indicates that the scale has good reliability; and a coefficient between 0.8 and 0.9 indicates very good reliability).

### Data analysis

SPSS 25.0, Bootstrap method, and SPSS macro program Process 3.5 plug-in (Model 6) were used for questionnaire data entry, statistical analysis, and chain mediating effect testing in the following order.

Firstly, the internal consistency of each variable was tested for reliability using Cronbach’s alpha coefficient.

Secondly, the Harman singleton test was used to test for the presence of common method bias in four variables: physical exercise, subjective well-being of older people, sense of meaning in life, and self-esteem.

Again, the mean, standard deviation and correlation coefficient of each variable were calculated using SPSS 25.0.

Again, covariance diagnosis is performed for possible multicollinearity.

Finally, regression analysis and the macro program Process 3.5 were used to conduct independent and chain mediating effect analysis of sense of meaning in life and self-esteem.

## Study results

### Common method deviation test

As the questionnaire was completed in an anonymous paper format, which is highly subjective, for possible cases of common method bias, this study conducted an unrotated exploratory factor analysis of the 31 variable entries in this study using the Harman one-way test. The results showed that four of the factors had eigenvalues greater than one and the first factor explained 36.02% of the variance, which was below the 40% threshold. So it can be inferred that there was no significant common method bias for the variables involved in this study.

### Correlation analysis of physical exercise, subjective well-being, sense of meaning in life, and self-esteem in older people

Physical exercise is significantly and positively related to subjective well-being, sense of meaning in life, and self-esteem, according to the findings in Table 2; subjective well-being is positively related to sense of meaning in life and self-esteem; and sense of meaning in life is positively related to self-esteem. This demonstrates that there is a significant positive correlation between the aforementioned variables, providing a foundation for future research to determine whether there is a chain mediating effect between the sense of meaning in life and self-esteem in the relationship between physical exercise and subjective well-being in the elderly.

**Table 2:** Correlation analysis of physical exercise, subjective well-being, sense of meaning in life and self-esteem in older people (*N* = 419).

Variables	*M*	SD	1	2	3	4
1 Physical exercise	33.69	31.43	1			
2 Subjective well-being of older people	37.20	12.62	0.295^**^	1		
3 Sense of meaning in life	23.44	6.75	0.351^**^	0.381^**^	1	
4 Self-esteem	8.37	2.75	0.348^**^	0.440^**^	0.434^**^	1

^**^p < 0.01.

### Multicollinearity test

To avoid problems with multicollinearity caused by significant correlations between all variables, which could result in unstable final results. The decision was made in this study to diagnose covariance using subjective well-being of older adults as the dependent variable and physical exercise, sense of meaning in life, and self-esteem as the independent variables, and to standardize each predictor variable. The results showed that each predictor variable’s tolerance value (0.846, 0.825, and 0.793) was greater than 0.1 and the VIF value (1.182, 1.212, 1, and 0.261) was less than 5. It can be concluded that the data did not have any multicollinearity issues and that it was suitable for further chain mediation effect testing.

### Analysis of the mediating effects of a sense of meaning in life and self-esteem

Using physical exercise as the independent variable, sense of meaning in life and self-esteem as mediating variables, and subjective well-being of older adults as the dependent variable, Hayes (2013) Bootstrap mediated effects analysis was conducted using Model 6 in the SPSS macro program Process 3.5 plug-in developed by Hayes. In this case, the replicate sample was 5,000 and the default confidence interval was 95%.

**Figure 2:**
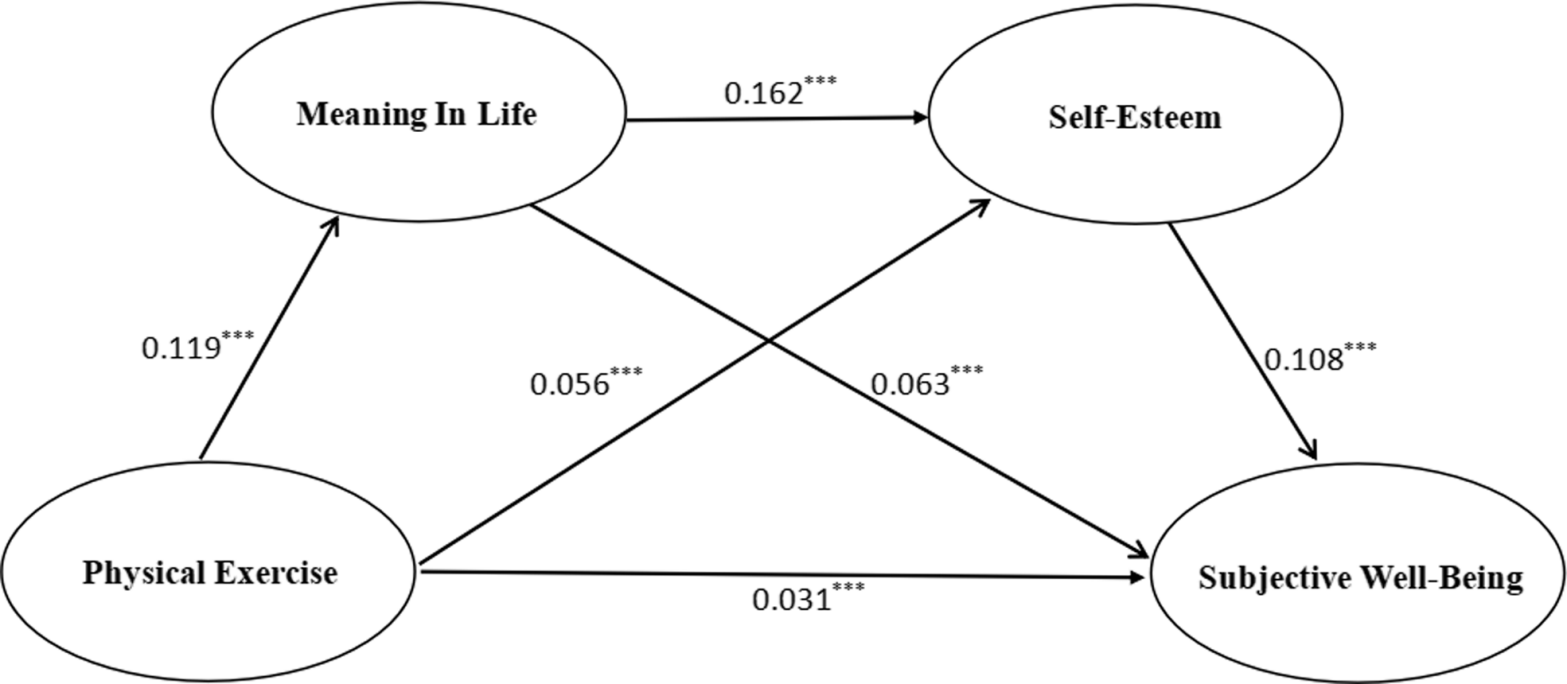
Diagram of the chain mediator model of a sense of meaning in life and self-esteem. ^***^p<0.001.

As the results in Table 3 and Figure 2 show, first, physical exercise not only directly and significantly and positively predicted subjective well-being (*β* = 0.149, *p* < 0.001), but also significantly and positively predicted sense of meaning in life (*β* = 0.119, *p* < 0.001) and self-esteem (*β* = 0.056, *p* < 0.001); second, sense of meaning in life significantly and positively predicted self-esteem (*β* = 0.162, *p* < 0.001) and subjective well-being (*β* = 0.063, *p* < 0.001); and third, self-esteem significantly predicted subjective well-being (*β* = 0.108, *p* < 0.001). This suggests that a sense of meaning in life and self-esteem play a full chain mediating effect between physical exercise and subjective well-being.

**Table 3:** Chain mediated model regression analysis of physical exercise, sense of meaning in life and self-esteem on subjective well-being of older people.

Regression equation		Overall fit index			Regression coefficient				
Resulting variables	Predictive variables	*R*	*R* ^2^	*F*	*β*	SE	*t*	LLCI	ULCI
sense of meaning in life	Physical exercise	0.295	0.087	39.824^***^	0.295	0.047	6.311^***^	0.203	0.387
Self-esteem	Physical exercise	0.455	0.207	54.387^***^	0.262	0.046	5.727^***^	0.172	0.352
	sense of meaning in life				0.303	0.046	6.637^***^	0.213	0.393
Subjective well-being	Physical exercise	0.549	0.301	59.688^***^	0.170	0.045	3.814^***^	0.082	0.258
	sense of meaning in life				0.289	0.045	6.404^***^	0.200	0.378
	Self-esteem				0.265	0.046	5.740^***^	0.174	0.355
Subjective well-being	Physical exercise	0.348	0.121	57.609^***^	0.348	0.046	7.590^***^	0.258	0.439

^***^p < 0.001.

As the results in Table 3 show, firstly, the mediation analysis yielded a total mediating effect of 0.1783, accounting for 51.18% of the total effect, and the 95% confidence interval (0.1177–0.2486) did not contain 0, indicating that the model with a chain of mediating variables of sense of life meaning and self-esteem held true and had some reliability. Secondly, by adding sense of life meaning and self-esteem to the relationship between physical exercise and subjective well-being, there were four pathways for the effect of physical exercise on subjective well-being. The direct effect of path 1 (physical exercise → subjective well-being) was 0.1701, with an effect ratio of 48.82%; the mediating effect of path 2 (physical exercise → Sense of meaning in life → self-esteem → subjective well-being) was 0.0237, with an effect ratio of 6.80%; the mediated effect of path 3 (physical exercise → Sense of meaning in life → subjective well-being) was 0.0854, with an effect of 24.51%; and the mediated effect for path 4 (physical exercise → self-esteem → subjective well-being) was 0.0692, with an effect ratio of 19.86%. The 95% confidence intervals of the above four pathways do not contain zero values, indicating that in the relationship between physical exercise and subjective well-being of the elderly, physical exercise, sense of meaning in life, and self-esteem can each independently influence subjective well-being, and sense of meaning in life and self-esteem can also jointly play a chain mediating effect, providing valid evidence for the above research hypothesis (Table 4).

**Table 4:** Chain mediation effect test between sense of meaning in life, self-esteem in physical exercise and subjective well-being.

Intermediary effect pathway	Standardized indirect effect values	Boot SE	95% confidence interval	Relative effects
Total effect	0.3484	0.0459	[0.2582–0.4386]	100%
Direct effects	0.1701	0.0446	[0.0824–0.2578]	48.82%
Physical exercise → Sense of meaning in life → Self-esteem → Subjective well-being	0.0237	0.0072	[0.0116–0.0396]	6.80%
Physical exercise → Sense of meaning in life → Subjective well-being	0.0854	0.0209	[0.0474–0.1331]	24.51%
Physical exercise → Self-esteem → Subjective well-being	0.0692	0.0176	[0.0374–0.1071]	19.86%
Total intermediary effect	0.1783	0.0339	[0.1177–0.2486]	51.18%

## Discussion

### The effect of physical exercise on subjective well-being of older people

Data analysis revealed that physical exercise predicted subjective well-being in older adults. This finding supports the study’s hypothesis and is consistent with the findings of Zheng research (Zheng, 2019). As a result, hypothesis H1 holds true, implying that the level of physical exercise is related to the level of subjective well-being of older adults, i.e., the higher the level of physical exercise rating, the higher the subjective well-being of older adults. Lower subjective well-being has been identified as one of the important factors contributing to high suicide rates, loneliness, and depression in older people (Wu et al., 2014; Bartlett and Arpin, 2019; Soósová et al., 2021). Physical exercise, as an external aid known to effectively counteract negative human emotions, will help to reduce the risk of suicide and depression, as well as loneliness (Sun and Stuart, 2002; Chen et al., 2010; Rogers et al., 2019). This can increase one’s subjective well-being. At the same time, a study also found that participating in physical exercise at a certain frequency and intensity helps older people strengthen their lower limb function while also improving their mental outlook and subjective well-being (Withall et al., 2014). The study also found that engaging in physical exercise at a certain frequency and intensity improved lower limb mobility as well as the mental outlook and subjective well-being of older people. Based on the preceding discussion, the hypothesis that physical exercise has a positive effect on the subjective well-being of older people is strengthened.

### The mediating role of a sense of meaning in life between physical exercise and subjective well-being of older people

The finding that a sense of meaning in life mediates the positive impact of physical exercise on subjective well-being in older adults supports Hypothesis 2. On the one hand, the study found that older adults who did not engage in physical exercise were more likely to experience anxiety, depression, helplessness, and other negative mental states that affect healthy physical and mental development, which lead individuals to lower their self-evaluation and generate a low sense of meaning in life through low self-evaluation, which ultimately inhibits the enhancement of subjective well-being. The lack of subjective well-being is exacerbated further by the increasing frequency of death cues in everyday life as a result of illness and declining body functions, particularly in old age (Binder and Buenstorf, 2018). On the other hand, this hypothesis is consistent with the self-worth theory, which states that in older people, a sense of meaning in life is a motivation to pursue and create value and meaning later in life, and that it is a powerful motivator to continue seeking newness and happiness in life. Older people who have a strong sense of meaning in life are more likely to envision a better future and take positive steps toward it, resulting in significant changes in their emotional feelings and lifestyles (Ju et al., 2013). This results in significant changes in emotional feelings and lifestyle choices, which contribute to an increase in subjective well-being. Conversely, older people who lack a sense of meaning in life and confidence in their future are more likely to exhibit negative feelings toward various things, which in turn impairs their subjective well-being. The findings of this study clarify the role of sense of meaning in life in enhancing subjective well-being and provide evidence of how physical exercise affects older people’s subjective well-being.

### The mediating role of self-esteem in the relationship between physical exercise and subjective well-being of older adults

Self-esteem is widely used in various sports psychology studies that measure post-exercise psychological representational characteristics as an important indicator of an individual’s psychological well-being (García et al., 2012). It has been proposed that long-term, consistent participation in physical exercise can address both low self-esteem and potential disease risk. A similar study in 246 older adults in China found a significant difference in self-esteem levels between those who did not exercise and those who did exercise, and that physical exercise not only directly but also indirectly influences the self-esteem levels of older adults (Yin et al., 2008), It is a significant factor influencing older people’s self-esteem. Furthermore, self-esteem is regarded as one of the most reliable predictors of subjective well-being (Diener and Diener, 1995; Diener, 2000). A review of previous research indicates that self-esteem is an important moderator in the relationship between physical exercise and subjective well-being. Square dancing, for example, as a form of self-organized physical exercise for Chinese older people, has changed their lifestyle and effectively improved their quality of life and sense of meaning in life (Shu, 2017). In this regard, Wu et al. (2019) conducted a study on the elderly population in square dance organizations. The study found that self-esteem could play a separate mediating role between physical exercise and subjective well-being, as well as a chain mediating role with organizational identity, which is consistent with the findings of this study. Therefore, the hypothesis H3 that self-esteem plays a mediating role between physical exercise and subjective well-being of older adults holds true.

### The chain mediating role of sense of meaning in life and self-esteem in the relationship between physical exercise and subjective well-being of older adults

Existing research suggests that having a sense of meaning in life can boost one’s self-esteem. Individual frustration, as Liu et al. (2020) pointed out in her study, is strongly influenced by a lack of a sense of meaning in life. Goal process theory suggests that value-expected goals guide changes in individuals’ value actions and perceptions, and when the results of actions do not match the expected goals, individuals develop strong negative emotions and engage in profound self-denial, resulting in individuals feeling more negative emotions and a decrease in self-esteem (Sang et al., 2019). This leads to a decrease in self-esteem. The elderly who have a higher sense of meaning in life can look forward to their future and deal positively and optimistically with all types of adverse events in their lives, which leads to good emotional feelings at all times, and when they face their lives in a positive state, their self-esteem will increase. As a result of the preceding research, this study investigated the mediating role of sense of meaning in life and self-esteem between physical exercise and subjective well-being in older adults and confirmed the chain mediating role of sense of meaning in life and self-esteem between physical exercise and subjective well-being in older adults, with hypothesis H4 being valid. Takkinen et al. (2001) discovered through long-term follow-up that older adults who regularly participated in physical exercise had a higher sense of meaning in life and that a good sense of meaning in life promotes higher self-esteem (Cao et al., 2019) and may further influence the perceived well-being of older adults including subjective well-being, optimistic experiences, and self-efficacy.

## Implications

Population aging is a significant social and public issue in China (Fang, 2021), Individuals, organizations, and nations will all face psychological and practical consequences. As the elderly population grows, it is important to pay attention to their physical and mental health. Physical exercise has long been recognized as an effective means of promoting individuals’ positive mental health (Wurm et al., 2014). This study reveals the impact of physical exercise on the subjective well-being of older people in China, as well as the mediating role of a sense of meaning in life and self-esteem, providing a foundation for positive psychological interventions for older people in order to effectively promote their psychological well-being.

This study further affirms the value of physical exercise on the basis of relevant research, and provides important support for enhancing the subjective well-being of older people through participation in physical exercise. In addition, we should encourage as many older people as possible to take part in regular and regular physical exercise.

Some scholars believe that good health problems, psychosocial competence, and social support can be effective in enhancing older people’s sense of meaning in life (Golovchanova et al., 2021). Other researchers have identified successful events experienced in life as an important factor in enhancing the level of self-esteem in older people (Rosi et al., 2019). Effectively supporting the existence of a chain mediating role of a sense of meaning in life and self-esteem in the relationship between physical exercise and subjective well-being in older adults, it opens up new pathways for interventions in the development of subjective well-being in older adults. Therefore, in the future life process, the subjective well-being of older people can be systematically enhanced through targeted participation in physical exercise and enhancing the level of sense of meaning in life and self-esteem.

## Conclusion

Physical exercise was found to be a significant predictor of subjective well-being in older people.

In older people, a sense of meaning in life and self-esteem levels can be mediated either individually or in a chain between physical exercise and subjective well-being. That is, the higher the level of physical exercise, the higher the sense of meaning in life and self-esteem of older people, which ultimately leads to an increase in subjective well-being. As a result, increasing older people’s sense of meaning in life and self-esteem levels will contribute to an increase in subjective well-being.

### Limitations and future research directions

This study provides a theoretical and practical reference for future research by explaining the complex psychological relationship between physical exercise participation, sense of meaning in life, self-esteem, and subjective well-being among Chinese older people. However, there are still some limitations in this study: (1) Because this is a cross-sectional study with many unexplored and uninvolved influencing factors, conducting a longitudinal study is an important task for consolidating this research in the next stage. (2) The independent and chain mediating role of sense of meaning in life and self-esteem between physical exercise and subjective well-being in the elderly is revealed in this study. However, due to China’s vast territory, there may be some variations in the exercise habits and methods of the elderly of different genders in different locations. As a result, in the next step, this study will consider moderating variables such as city, age, gender, income, marital status, and education level. (3) This study examined the relationship between the intensity, duration, and frequency of physical exercise and subjective well-being of older people. However, as the type of exercise program may affect the findings, the next study will consider the effects of exercise intensity, duration, and frequency of participation in different types of programs on the subjective well-being of older people based on the distinction between objective evaluation and subjective perception, as well as the mediating effects of related intrinsic mechanisms.

## Data availability statement

The raw data supporting the conclusions of this article will be made available by the authors, without undue reservation.

## Ethics statement

The studies involving human participants were reviewed and approved by Chengdu Sport University. The patients/participants provided their written informed consent to participate in this study.

## Author contributions

RC was responsible for analyzing the experimental data and writing and revising in English. G-DH and P-CW were responsible for collecting, collating, and analyzing the experimental data. Y-FL was responsible for conceptualizing and checking and revising the article. All authors contributed to the article and approved the submitted version.

## Funding

This work was supported by the Chengdu Institute of Sports Research and Innovation Team Grant No. CXTD201801.

## Conflict of interest

The authors declare that the research was conducted in the absence of any commercial or financial relationships that could be construed as a potential conflict of interest.

## Publisher’s note

All claims expressed in this article are solely those of the authors and do not necessarily represent those of their affiliated organizations, or those of the publisher, the editors and the reviewers. Any product that may be evaluated in this article, or claim that may be made by its manufacturer, is not guaranteed or endorsed by the publisher.
